# Transient Gel Diffusiophoresis of a Spherical Colloidal Particle

**DOI:** 10.3390/mi16030266

**Published:** 2025-02-26

**Authors:** Hiroyuki Ohshima

**Affiliations:** Faculty of Pharmaceutical Sciences, Tokyo University of Science, 2641 Yamazaki, Noda 278-8510, Japan; ohshima@rs.noda.tus.ac.jp

**Keywords:** transient gel diffusiophoresis, gel diffusiophoresis, transient diffusiophoresis, diffusiophoresis, spherical colloidal particle, gel

## Abstract

A general theory is presented to analyze the time-dependent, transient diffusiophoresis of a charged spherical colloidal particle in an uncharged gel medium containing a symmetrical electrolyte when an electrolyte concentration gradient is suddenly applied. We derive the inverse Laplace transform of an approximate expression for the relaxation function *R*(*t*), which describes the time-course of the ratio of the diffusiophoretic mobility of a weakly charged spherical colloidal particle, possessing a thin electrical double layer, to its steady-state diffusiophoretic mobility. The relaxation function depends on the mass density ratio of the particle to the electrolyte solution, the particle radius, the Brinkman screening length, and the kinematic viscosity. However, it does not depend on the type of electrolyte (e.g., KCl or NaCl), which affects only the steady-state gel diffusiophoretic mobility. It is also found that the expression for the relaxation function in transient gel diffusiophoresis of a weakly charged spherical colloidal particle with a thin electrical double layer takes the same form as that for its transient gel electrophoresis.

## 1. Introduction

The zeta potential of colloidal particles is a key parameter in the Derjaguin-Landau-Verwey-Overbeek (DLVO) theory of colloidal suspension stability [1,2,3,4,5,6,7,8,9,10,11,12,13]. It can be determined through electrokinetic techniques, including electrophoresis and diffusiophoresis. Both electrophoresis and diffusiophoresis are fundamental electrokinetic phenomena that govern the motion of charged colloidal particles in liquid media. Electrophoresis involves particle migration under an externally applied electric field, while diffusiophoresis is driven by an electrolyte concentration gradient.

Extensive theoretical studies have explored diffusiophoresis in various colloidal systems, including rigid spheres [14,15,16,17,18,19,20,21,22,23,24,25,26,27] and soft particles with polyelectrolyte coatings [13,28]. One of the key applications of diffusiophoresis is in drug delivery systems (DDS), where electrolyte concentration gradients can guide particles toward specific regions or tissues, enabling more precise and efficient therapeutic interventions. Additionally, as diffusiophoresis operates without external energy input, it is particularly advantageous in microfluidic environments.

The electrokinetic behavior of colloidal particles is commonly examined in free-solution environments, where colloidal particles migrate in an electrolyte solution without spatial constraints. However, electrokinetics in porous media, such as gel electrophoresis, has both theoretical and practical significance [29,30,31]. When particles travel through the pores of a gel matrix, they experience two primary interactions: (i) short-range steric effects from particle-gel friction and (ii) long-range hydrodynamic interactions. In densely cross-linked gels, where pore sizes are smaller than the particles, steric effects dominate. Conversely, in dilute gels, where the pore size is significantly larger than the particle, long-range hydrodynamic interactions become more influential. The polymer network within a gel can be treated as a porous structure filled with an electrolyte solution, following the Brinkman-Debye-Bueche model [32,33], where polymer segments act as friction centers within the fluid. Using this framework, numerous theoretical studies have explored the diffusiophoresis of rigid particles [34,35,36,37,38,39,40,41,42,43] and soft particles [13,44,45,46,47,48,49].

Sambamoorthy and Chu [50] and Bhaskar and Bhattacharyya [51] developed a theoretical framework for the diffusiophoresis of charged spherical particles in porous gels. Their studies introduced governing equations for particle mobility in gel environments and outlined numerical methods to evaluate these mobilities. Our recent research [52] built upon these foundational works, further generalizing the theoretical framework to derive a more comprehensive expression for particle motion in gel diffusiophoresis.

Despite growing recognition of diffusiophoresis, most prior studies have concentrated on steady-state conditions, where the electrolyte concentration gradient remains unchanged over time, leading to a constant particle velocity. While this steady-state behavior is well understood, transient diffusiophoresis—where particles respond to a suddenly imposed concentration gradient—remains largely unexplored. Understanding this transient response is critical for practical applications, as real-world scenarios often involve abrupt environmental changes rather than perfectly stable conditions. This knowledge gap contrasts with transient electrophoresis, which has been extensively studied. In transient electrophoresis, researchers have analyzed how colloidal particles accelerate after an electric field is suddenly applied, eventually reaching a steady-state velocity. Foundational studies by Morrison [53,54] and Ivory [55,56], later expanded by Keh and collaborators [57,58,59,60,61,62,63,64] as well as others [65,66,67,68,69,70], have provided detailed insights into transient electrophoresis across various particle types [53,54,55,56,57,58,59,60,61,62,63,64,65,66,67,68,69,70], including rigid spheres [53,56,58,59,60,64,66,69], cylinders [54,61,62,67], porous particles [63], and soft particles [15]. Research has also addressed transient gel electrophoresis, investigating time-dependent particle behavior in polymer gel matrices [71,72,73,74,75,76].

However, research on transient diffusiophoresis, in which an electrolyte concentration gradient is suddenly imposed and the resulting particle dynamics are examined, remains scarce in the literature. Our recent theoretical analysis [77] established a general framework for time-dependent transient diffusiophoresis in a free electrolyte solution, focusing on weakly charged spherical colloidal particles with a thin electric double layer.

In the present paper, we further extend our recent work on transient free-solution diffusiophoresis [77] to examine a previously unexplored type of transient electrokinetics—transient gel diffusiophoresis of a spherical colloidal particle. Investigating this phenomenon is essential for understanding particle transport in complex environments, such as porous media and biological systems, where electrolyte concentration gradients play a crucial role.

## 2. Theory

**Figure 1:**
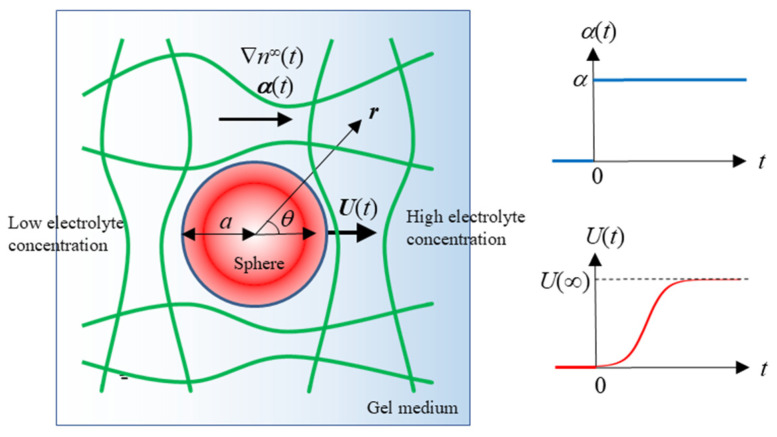
Transient gel diffusiophoresis of a spherical colloidal particle with radius *a* moving with a transient diffusiophoretic velocity ***U***(*t*) in an electrolyte concentration gradient field ∇*n^∞^*(*t*) or ***a***(*t*).

Consider a charged spherical colloidal particle with radius *a*, relative permittivity *ε*_p_, and mass density *ρ*_p_, carrying zeta potential *ζ* in an uncharged polymer gel medium containing an electrolyte solution with viscosity *η* and relative permittivity *ε*_r_. The Brinkman-Debye-Bueche continuum medium [32,33] is applied, where polymer segments act as resistance centers dispersed throughout the gel medium, generating frictional forces on the liquid as it moves through the gel medium. The gel medium is treated as a homogeneous continuum medium containing electrolyte ions with a density of *ρ*_el_(***r***) at position ***r***. The electrolyte is assumed to be a symmetrical type with valence *z*, allowing for different ionic drag coefficients *λ*_+_ for cations and *λ*_−_ for anions. A spherical coordinate system (*r*, *θ*, *ϕ*) is employed with its origin at the center of the particle (Figure 1). The concentrations (number densities) of cations and anions in the electrolyte solution at position ***r*** and time *t* are denoted as *n*_+_(***r***, *t*) and *n*_−_(***r***, *t*), respectively, while *n^∞^* represents their concentrations beyond the electrical double layer surrounding the particle.

Suppose that at time *t* = 0, a step electrolyte concentration gradient ∇*n^∞^*(*t*) is abruptly applied along the polar axis *θ* = 0, such that

(1) $$\nabla n^{\infty }\left(t\right)=\left\{\begin{array}{cc} 0, & \;\;\;\;t=0\\ \nabla n^{\infty }, & \;\;\;\;t>0 \end{array}\right.$$

We now define a vector ***α***(*t*) as

(2) $$\alpha \left(t\right)=\left(\alpha \left(t\right)\cos\theta ,-\alpha \left(t\right)\sin\theta ,0\right)=\frac{kT}{ze}\nabla \ln(n^{\infty }\left(t\right))$$

with

(3) $$\alpha \left(t\right)=\left\{\begin{array}{c} 0,\;\;\;\;\;\;\;\;\;\;\;\;\;\;\;t=0\;\\ \alpha ,\;\;\;\;\;\;\;\;\;\;\;\;\;\;\;t>0 \end{array}\right.$$

and

(4) $$\alpha =\left(\alpha \cos\theta ,-\alpha \sin\theta ,0\right)=\frac{kT}{ze}\nabla \ln(n^{\infty })$$

Here, *α*(*t*) and *α*, respectively, denote the magnitudes of ***α***(*t*) and ***α***, *e* is the elementary charge, *k* is the Boltzmann constant, and *T* is the absolute temperature. Then, the particle starts moving with a diffusiophoretic velocity ***U***(*t*) in the direction parallel to ∇*n^∞^*(*t*) or ***α***(*t*) (Figure 1). The diffusiophoretic mobility *μ*(*t*) is defined as

(5) $$U\left(t\right)=\mu \left(t\right)\alpha \left(t\right)$$

We consider the following assumptions: (i) The liquid is treated as incompressible. (ii) The applied electrolyte concentration gradient field ***α***(*t*) is sufficiently weak such that both the particle velocity ***U***(*t*) and the liquid velocity ***u***(***r***, *t*) remain proportional to ***α***(*t*). This allows us to neglect the terms involving the square of the liquid velocity in the Navier-Stokes equation. (iii) The slipping plane, defined as the plane where the liquid velocity ***u***(***r***, *t*) at position ***r*** (*r*, 0, 0) relative to the particle velocity is zero, coincides with the particle surface at *r* = *a*. Here, *r* represents the radial distance from the particle center. (iv) Electrolyte ions cannot penetrate the particle surface. (v) In the absence of ***α***(*t*), the electrolyte ion distribution follows the Boltzmann distribution, and the equilibrium potential *Ψ*^(0)^(*r*) is governed by the Poisson-Boltzmann equation. (vi) The relative permittivity *ε*_p_ of the particle is much lower than that of the surrounding electrolyte solution *ε*_r_ (*ε*_p_ « *ε*_r_), making *ε*_p_ nearly negligible. (vii) The Brinkman-Debye-Bueche continuum medium model [32,33] is employed, in which the polymer segments are regarded as resistance centers, exerting frictional forces with a frictional coefficient *γ* on the liquid flowing through the gel medium.

Under assumptions (i) through (vii), we derive the following fundamental electrokinetic equations governing the liquid velocity ***u***(***r***, *t*) = (*u_r_*(***r***, *t*), *u_θ_*(***r***, *t*), 0) at position ***r*** and time *t*, as well as the velocity ***v***_+_(***r***, *t*) of cations and ***v***_−_(***r***, *t*) of anions, which are similar to those for transient gel electrophoesis and transient diffusiophoesis of a sphere [75,77]:

(6) $$\rho_{o}\frac{\partial }{\partial t}\left\{u\left(r,t\right)+U\left(t\right)\right\}+\eta \nabla \times \nabla \times u\left(r,t\right)+\nabla p\left(r,t\right)+\rho_{\mathrm{el}}\left(r,t\right)\nabla \psi \left(r,t\right)+\gamma \left\{u\left(r,t\right)+U\left(t\right)\right\}=0$$

(7) $$\nabla \cdot u\left(r,t\right)=0$$

(8) $$v_{\pm }\left(r,t\right)=u\left(r,t\right)-\frac{1}{\lambda_{\pm }}\nabla \mu_{\pm }\left(r,t\right)$$

(9) $$\frac{\partial n_{\pm }\left(r,t\right)}{\partial t}+\nabla \cdot \left\{n_{\pm }\left(r,t\right)v_{\pm }\left(r,t\right)\right\}=0$$

(10) $$\rho_{\mathrm{el}}\left(r,t\right)=ze\left\{n_{+}\left(r,t\right)-n_{-}\left(r,t\right)\right\}$$

(11) $$\mu_{\pm }\left(r,t\right)=\mu_{\pm }^{o}\pm ze\psi \left(r,t\right)+kT\ln\left[n_{\pm }\left(r,t\right)\right]$$

(12) $$\Delta \psi \left(r,t\right)=-\frac{\rho_{\mathrm{el}}\left(r,t\right)}{\varepsilon_{r}\varepsilon_{0}}$$

(13) $$\frac{4\pi a^{3}}{3}\rho_{p}\frac{dU\left(t\right)}{dt}=F_{H}\left(t\right)+F_{E}\left(t\right)$$

Here, *ε*_0_ is the permittivity of a vacuum, *p*(***r***, *t*) is the pressure, *ρ*_el_(***r***, *t*) represents the charge density (given by Equation (10)), and *Ψ*(***r***, *t*) represents the electric potential. Equations (6) and (7) are the Navier-Stokes equation and the continuity equation for incompressible flow ***u***(***r***, *t*) (condition (i)), where the term *ρ*_0_(***u***·∇)***u*** is omitted (condition (ii)). The term involving the frictional coefficient *γ* in Equation (6) represents the frictional force exerted on the liquid flow by the polymer segments in the polymer gel medium. The presence of the particle velocity ***U***(*t*) in Equation (6) results from the fact that the particle is chosen as the reference frame for the coordinate system. Equation (8) describes the flow of the electrolyte ions as driven by both the fluid motion ***u***(***r***, *t*) and the gradient of the electrochemical potentials of cations *μ*_+_(***r***, *t*) and anions *μ*_−_(***r***, *t*), which are given by Equation (11) with $\mu_{\pm }^{o}$ being a constant. Equation (9) is the continuity equation for the cations and anions, while Equation (12) is the Poisson equation. Equation (13) is the equation of motion of the particle, in which ***F***_H_(*t*) and ***F***_E_(*t*) are, respectively, the hydrodynamic and electric forces acting on the particle and are given by

(14) $$F_{H}\left(t\right)=\int_{0}^{\pi }\left[\left\{-p\left(r,t\right)+2\eta \frac{\partial u_{r}\left(r,t\right)}{\partial r}\right\}\cos\theta -\eta \left\{\frac{1}{r}\frac{\partial u_{r}\left(r,t\right)}{\partial \theta }+\frac{\partial u_{\theta }\left(r,t\right)}{\partial r}-\frac{u_{\theta }\left(r,t\right)}{r}\right\}\sin\theta \right]2\pi a^{2}\sin\theta d\theta \frac{\alpha }{\alpha }$$

$$F_{E}\left(t\right)=\varepsilon_{r}\varepsilon_{0}\int_{0}^{\pi }\left[\frac{\partial \psi \left(r,t\right)}{\partial r}\left(\frac{\partial \psi \left(r,t\right)}{\partial r}\cos\theta -\frac{1}{r}\frac{\partial \psi \left(r,t\right)}{\partial \theta }\sin\theta \right)\right.$$

(15) $$-\frac{1}{2}\left.\left\{{\left(\frac{\partial \psi \left(r,t\right)}{\partial r}\right)}^{2}+{\left(\frac{1}{r}\frac{\partial \psi \left(r,t\right)}{\partial \theta }\right)}^{2}\right\}\cos\theta \right]2\pi a^{2}\sin\theta d\theta \frac{\alpha }{\alpha }$$

The initial conditions at *t* = 0 and the boundary conditions at the particle surface (*r* = a) and far from it (*r* → ∞) must be satisfied as [77]

(16) $$U\left(t\right)=0\;\mathrm{at}\;\;t=0$$

(17) $$u\left(r,t\right)=0\;\mathrm{at}\;\;t=0$$

(18) $$u\left(r,t\right)=0\;\;\;\mathrm{at}\;r=a$$

(19) $$u\left(r,t\right)\to -U\left(t\right)+\frac{a^{3}\left(\rho_{p}-\rho_{0}\right)}{3\rho_{0}r^{3}}\;\left[U\left(t\right)-3\left\{\left(U\left(t\right)\cdot \hat{r}\right)\hat{r}\right\}\right]\;\mathrm{as}\;r\to \infty$$

(20) $$v_{\pm }\left(r,t\right)\cdot \hat{n}=0\;\;\mathrm{at}\;r=a$$

where $\hat{r}$ = ***r***/*r* and $\hat{n}$ is the unit normal vector pointing outward from the particle surface. Equations (16) and (17) imply that both the particle and the liquid are at rest at time *t* = 0. Equation (18) implies that the slipping plane, where ***u***(***r***, *t*) = **0**, is located on the particle surface (condition (iii)). Equations (19) and (20) follow from Equation (13) and condition (iv), respectively [77].

For a weak field ***α***(*t*), the deviations *δn_±_*(***r***, *t*), *δΨ*(***r***, *t*), *δμ_±_*(***r***, *t*), and *δρ*_el_(***r***, *t*) of *n_±_*(***r***, *t*), *Ψ*(***r***, *t*), *μ_±_*(***r***, *t*), and *ρ*_el_(***r***, *t*) from their equilibrium values (i.e., those in the absence of the electrolyte concentration gradient field ***α***(*t*)) are small. We may thus write

(21) $$n_{\pm }\left(r,t\right)=n_{\pm }^{\left(0\right)}\left(r\right)+\delta n_{\pm }\left(r,t\right)$$

(22) $$\psi \left(r,t\right)=\psi^{\left(0\right)}\left(r\right)+\delta \psi \left(r,t\right)$$

(23) $$\mu_{\pm }\left(r,t\right)=\mu_{\pm }^{\left(0\right)}+\delta \mu_{\pm }\left(r,t\right)$$

(24) $$\rho_{\mathrm{el}}\left(r,t\right)=\rho_{\mathrm{el}}^{\left(0\right)}\left(r\right)+\delta \rho_{\mathrm{el}}\left(r,t\right)$$

where the superscript (0) denotes the equilibrium quantities in the absence of ***α***(*t*), which depend only on *r*, and $\mu_{\pm }^{\left(0\right)}$ is constant and independent of *r*.

Let us assume that the equilibrium concentration $n_{\pm }^{\left(0\right)}\left(r\right)$ obeys the Boltzmann distribution, and the equilibrium electric potential *Ψ*^(0)^(*r*) satisfies the Poisson-Boltzmann equation (condition (v)), namely:

(25) $$n_{\pm }^{\left(0\right)}\left(r\right)=n^{\infty }e^{\mp y\left(r\right)}$$

(26) $$\Delta y\left(r\right)=\kappa^{2}\sin hy\left(r\right)$$

with

(27) $$y\left(r\right)=\frac{ze\psi^{\left(0\right)}\left(r\right)}{kT}$$

(28) $$\kappa =\sqrt{\frac{2z^{2}e^{2}n^{\infty }}{\varepsilon_{r}\varepsilon_{0}kT}}$$

where *y*(*r*) is the scaled equilibrium electric potential, and *ĸ* is the Debye-Hückel parameter (where 1/*ĸ* is the Debye length). The following boundary conditions for *n*_±_^(0)^(*r*) and *ψ*^(0)^(*r*) must be satisfied:

(29) $$n_{\pm }^{\left(0\right)}\left(r\right)\to n^{\infty }\;\mathrm{as}\;r\;\to \infty$$

(30) $$\psi^{\left(0\right)}\left(r\right)\to 0\;\mathrm{as}\;r\;\to \infty$$

(31) $$\psi^{\left(0\right)}\left(a\right)=\zeta$$

Under an applied electrolyte concentration gradient field ∇*n^∞^*(*t*) or ***α***(*t*), the boundary condition of *δn*_±_(***r***, *t*) at distances far from the particle is expressed as

(32) $$\delta n_{\pm }\left(r,t\right)\to \left|\nabla n^{\infty }\left(t\right)\right|r\cos\theta =\frac{zen^{\infty }}{kT}\alpha \left(t\right)r\cos\theta \;\mathrm{as}\;r\;\to \infty$$

The far-field boundary condition for *δΨ*(***r***, *t*) can be derived as follows. The ionic flows ***v***_±_(***r*,** *t*) induced by ***α***(*t*) generate a macroscopic electric field ***E***(*t*), known as the diffusion potential field, which nullifies the net electric current so that *δΨ*(***r***, *t*) does not vanish as *r*→∞. The electric current density ***i***(***r***, *t*) at position ***r*** and time *t* is given by

(33) $$i\left(r,t\right)=ze\left\{n_{+}\left(r,t\right)v_{+}\left(r,t\right)-n_{-}\left(r,t\right)v_{-}\left(r,t\right)\right\}$$

By substituting Equations (8), (10), (21), and (23) into Equation (33) and ignoring the products of small quantities ***u***(***r***, *t*), *δn*_±_(***r***, *t*), and *δμ*_±_(***r***, *t*), we have

(34) $$i\left(r,t\right)=\rho_{\mathrm{el}}^{\left(0\right)}\left(r\right)u\left(r,t\right)-ze\left\{\frac{n_{+}^{\left(0\right)}\left(r\right)}{\lambda_{+}}\nabla \delta \mu_{+}\left(r,t\right)-\frac{n_{-}^{\left(0\right)}\left(r\right)}{\lambda_{-}}\nabla \delta \mu_{-}\left(r,t\right)\right\}$$

With the help of the relation

(35) $$\delta \mu_{\pm }\left(r,t\right)=\pm ze\delta \psi \left(r,t\right)+kT\frac{\delta n_{\pm }\left(r,t\right)}{n_{\pm }^{\left(0\right)}\left(r\right)}$$

which is derived from Equations (11) and (19)–(21), Equation (32) reduces to

$$i\left(r,t\right)=\rho_{\mathrm{el}}^{\left(0\right)}\left(r\right)u\left(r,t\right)-{\left(ze\right)}^{2}\left\{\frac{n_{+}^{\left(0\right)}\left(r\right)}{\lambda_{+}}+\frac{n_{-}^{\left(0\right)}\left(r\right)}{\lambda_{-}}\right\}\nabla \delta \psi \left(r,t\right)$$

(36) $$-zekT\left\{\frac{n_{+}^{\left(0\right)}\left(r\right)}{\lambda_{+}}\nabla \ln\left[n_{+}\left(r,t\right)\right]-\frac{n_{-}^{\left(0\right)}\left(r\right)}{\lambda_{-}}\nabla \ln\left[n_{-}\left(r,t\right)\right]\right\}$$

Beyond the electrical double layer around the particle (*r*→*∞*),

(37) $$n_{\pm }^{\left(0\right)}\left(r\right)\;\to n^{\infty },\rho_{\mathrm{el}}^{\left(0\right)}\left(r\right)\;\to 0,\nabla \psi \left(r,t\right)\to -E\left(t\right),\;\mathrm{and}\;\;\nabla \ln\left[n_{\pm }\left(r,t\right)\right]\to \nabla \ln\left(n^{\infty }\right)\;\mathrm{as}\;r\to \infty$$

and thus Equation (32) becomes

(38) $$i\left(r,\;t\right)\to {\left(ze\right)}^{2}n^{\infty }\left(\frac{1}{\lambda_{+}}+\frac{1}{\lambda_{-}}\right)E-zekTn^{\infty }\left(\frac{1}{\lambda_{+}}-\frac{1}{\lambda_{-}}\right)\nabla \ln\left(n^{\infty }\right)\;\mathrm{as}\;r\to \infty$$

where *E*(*t*) is the magnitude of ***E***(*t*). Since ***i***(***r***, *t*) must be zero far from the particle (*r*→∞), we find from Equation (32) that

(39) $$E\left(t\right)=\beta \alpha \left(t\right)$$

Here *β* is defined as

(40) $$\beta =\frac{1/\lambda_{+}-1/\lambda_{-}}{1/\lambda_{+}+1/\lambda_{-}}=-\frac{\lambda_{+}-\lambda_{-}}{\lambda_{+}+\lambda_{-}}$$

We thus obtain

(41) $$\delta \psi \left(r,t\right)\to -\beta \alpha \left(t\right)r\cos\theta \;\mathrm{as}\;r\to \infty$$

By combining Equations (32), (35), and (41), we obtain the following far-field boundary condition for *δμ*_±_(***r***, *t*):

(42) $$\delta \mu_{\pm }\left(r\right)\to ze\left(1\mp \beta \right)\alpha \left(t\right)r\cos\theta \;\mathrm{as}\;r\;\to \infty$$

By inserting Equations (19)–(22) into Equation (6) and neglecting the products of small quantities ***u***(***r***, *t*), *δn*_±_(***r***, *t*), *δΨ*(***r***, *t*), and *δμ*_±_(***r***, *t*) (condition (ii)), we obtain

(43) $$\rho_{0}\frac{\partial }{\partial t}\nabla \times u\left(r,t\right)+\eta \nabla \times \nabla \times \nabla \times u\left(r,t\right)+\gamma \nabla \times u\left(r,t\right)=\nabla \delta \mu_{+}\left(r,t\right)\times \nabla n_{+}^{\left(0\right)}\left(r\right)+\nabla \delta \mu_{-}\left(r,t\right)\times \nabla n_{-}^{\left(0\right)}\left(r\right)$$

and from Equations (8) and (9)

(44) $$\frac{\partial }{\partial t}\delta n_{\pm }\left(r,t\right)+\nabla \cdot \left\{n_{\pm }^{\left(0\right)}\left(r\right)u\left(r,t\right)-\frac{1}{\lambda_{\pm }}n_{\pm }^{\left(0\right)}\left(r\right)\nabla \delta \mu_{\pm }\left(r,t\right)\right\}=0$$

Furthermore, symmetry considerations allow us to express [29,36]:

(45) $$u\left(r,t\right)=\left(-\frac{2}{r}h\left(r,t\right)\alpha \left(t\right)\cos\theta ,\;\frac{1}{r}\frac{\partial }{\partial r}\left(rh\left(r,t\right)\right)\alpha \left(t\right)\sin\theta ,\;0\right)$$

(46) $$\delta \mu_{\pm }\left(r,\;t\right)=\mp ze\phi_{\pm }\left(r,t\right)\alpha \left(t\right)\cos\theta$$

(47) $$\delta \psi \left(r,\;t\right)=-Y\left(r,t\right)\alpha \left(t\right)\cos\theta$$

where *h*(*r*, *t*), *ϕ_±_*(*r*, t), and *Y*(*r*, *t*) are functions of *r* and *t*. By substituting Equations (45)–(47) into Equations (36) and (37), we arrive at the following equations for *h*(*r*, *t*), *ϕ*_±_(*r*, t), and *Y*(*r*, *t*)

(48) $$L\left[Lh\left(r,t\right)-\lambda^{2}h\left(r,t\right)-\frac{1}{\nu }\frac{\partial h\left(r,t\right)}{\partial t}\right]=G\left(r,t\right)$$

(49) $$L\phi_{\pm }\left(r,t\right)-\frac{\lambda_{\pm }}{kT}\frac{\partial }{\partial t}\left\{\phi_{\pm }\left(r,t\right)-Y\left(r,t\right)\right\}=\pm \frac{dy\left(r\right)}{dr}\left\{\frac{d\phi_{\pm }\left(r,t\right)}{dr}\mp \frac{2\lambda_{\pm }}{e}\frac{h\left(r,t\right)}{r}\right\}$$

(50) $$LY\left(r,t\right)=\frac{z^{2}e^{2}}{\varepsilon_{r}\varepsilon_{0}kT}\left[n_{+}^{\left(0\right)}\left(r\right)\left\{Y\left(r,t\right)-\phi_{+}\left(r,t\right)\right\}+n_{-}^{\left(0\right)}\left(r\right)\left\{Y\left(r,t\right)-\phi_{-}\left(r,t\right)\right\}\right]$$

with

(51) $$\lambda =\sqrt{\frac{\gamma }{\eta }}$$

Here *λ* is the Brinkman parameter (where 1/*λ* is the Brinkman screening length), and *L* is a Differential operator defined as

(52) $$L=\frac{\partial }{\partial r}\frac{1}{r^{2}}\frac{\partial }{\partial r}r^{2}=\;\frac{\partial^{2}}{\partial r^{2}}+\frac{2}{r}\;\frac{\partial }{\partial r}-\frac{2}{r^{2}}\;\;$$

*G*(*r*, *t*) is defined as

(53) $$G\left(r,t\right)=-\frac{zen^{\infty }}{\eta r}\frac{dy}{dr}\left\{e^{-y}\phi_{+}\left(r,t\right)+e^{y}\phi_{-}\left(r,t\right)\right\}$$

and

(54) $$\nu =\frac{\eta }{\rho_{o}}$$

is the kinematic viscosity. The initial and boundary conditions, given by Equations (16)–(20) and (42), can be rewritten in terms of *h*(*r*, *t*), *ϕ_±_*(*r*, *t*), and *Y*(*r*, *t*) as follows [77]:

(55) $$h\left(r,\;t\right)=\frac{\partial h\left(r,\;t\right)}{\partial r}=0\;\;\mathrm{at}\;\;t=0$$

(56) $$h\left(r,\;t\right)=\frac{\partial h\left(r,\;t\right)}{\partial r}=0\;\;\mathrm{at}\;r=a$$

(57) $$h\left(r,t\right)\to \frac{\mu \left(t\right)}{2}r+\frac{a^{3}\left(\rho_{p}-\rho_{0}\right)}{3\rho_{0}r^{2}}\mu \left(t\right)\;\;\mathrm{as}\;r\to \infty$$

(58) $$\phi_{\pm }\left(r,t\right)\to \left(\mp 1+\beta \right)r\;\mathrm{as}\;r\to \infty$$

(59) $$\frac{\partial \phi_{\pm }\left(r,t\right)}{\partial r}=0\;\;\mathrm{at}\;\;r=a$$

(60) $$Y\left(r,t\right)\to \beta r\;\mathrm{as}\;r\to \infty$$

The transient gel diffusiophoretic mobility *μ*(*t*) (defined by Equation (5)) can be obtained from Equation (45), viz.:

(61) $$\mu \left(t\right)=\frac{U\left(t\right)}{\alpha }=2\underset{r\to \infty }{\lim}\frac{h\left(r,\;t\right)}{r}$$

Here, *h*(*r*, *t*) is the solution to Equation (48), which can be most conveniently solved using the Laplace transformation with respect to time *t*. The Laplace transforms $\hat{h}\left(r,s\right)$, $\hat{G}\left(r,s\right)$, and $\hat{\mu }\left(s\right)$ of *h*(*r*, *t*), *G*(*r*, *t*), and *μ*(*t*), respectively, are given by:

(62) $$\hat{h}\left(r,s\right)=L\left[h\left(r,t\right)\right]=\int_{0}^{\infty }h\left(r,t\right)e^{-st}dt$$

(63) $$\hat{G}\left(r,s\right)=L\left[G\left(r,t\right)\right]=\int_{0}^{\infty }G\left(r,t\right)e^{-st}dt$$

(64) $$\hat{\mu }\left(s\right)=L\left[\mu \left(t\right)\right]=\int_{0}^{\infty }\mu \left(t\right)e^{-st}dt$$

From Equations (60), (61), and (73), we obtain the following general expression for $\hat{\mu }\left(s\right)$:

(65) $$\hat{\mu }\left(s\right)=2\underset{r\to \infty }{\lim}\frac{\hat{h}\left(r,s\right)}{r}$$

The Laplace transform of Equation (48) thus gives

(66) $$L\left[L\hat{h}\left(r,s\right)-\lambda^{2}\hat{h}\left(r,s\right)-\frac{s}{\nu }\hat{h}\left(r,s\right)\right]=\hat{G}\left(r,s\right)\;$$

which can be solved to yield

(67) $$\hat{\mu }\left(s\right)=-\frac{2\int_{a}^{\infty }\left\{1+a\sqrt{\lambda^{2}+\frac{s}{\nu }}+\frac{a^{2}}{3}\left(\lambda^{2}+\frac{s}{\nu }\right)-\left(1+\sqrt{\lambda^{2}+\frac{s}{\nu }}\;r\right)e^{-\sqrt{\lambda^{2}+\frac{s}{\nu }}\;\left(r-a\right)}-\frac{r^{3}}{3a}\left(\lambda^{2}+\frac{s}{\nu }\right)\right\}\hat{G}\left(r,s\right)dr\;}{3\left(\lambda^{2}+\frac{s}{\nu }\right)\left\{1+a\sqrt{\lambda^{2}+\frac{s}{\nu }}+\frac{a^{2}}{9}\left(\lambda^{2}+\frac{s}{\nu }\right)+\frac{2a^{2}\rho_{p}s}{9\nu \rho_{0}}\right\}}$$

The transient gel diffusiophoretic mobility *μ*(*t*) can be obtained by applying the numerical inverse Laplace transform to Equation (67). It is possible to derive an approximate expression applicable for the practically important case of a weakly charged spherical particle with a thin electrical double layer (*ĸa* » 1) and a negligibly small *ε*_p_ (*ε*_p_ « *ε*_0_). In this case, it can be shown that *Y*(*r*) becomes equal to *ϕ*_±_(r), simplifying the solution of Equation (49) for *ϕ*_±_(r), as shown below. For the low zeta potential case, by solving Equations (48)–(50), *G*(*r*, *t*) is found to be independent of *t* so that *G*(*r*, *t*) can be rewritten as *G*(*r*) and $\hat{G}\left(r,s\right)$ can be expressed as

(68) $$\hat{G}\left(r,s\right)=\frac{G\left(r\right)}{s}=-\frac{2zen^{\infty }}{\eta }\frac{dy}{dr}\left[\left(1+\frac{a^{3}}{2r^{3}}\right)\left\{\beta +y+\frac{1}{3}\int_{a}^{\infty }\frac{dy}{dr}\left(1-\frac{a^{3}}{r^{3}}\right)dr\right\}-\frac{1}{3}\int_{a}^{r}\frac{dy}{dx}\left(1-\frac{x^{3}}{r^{3}}\right)\left(1-\frac{a^{3}}{x^{3}}\right)dx\right]$$

where the low-zeta potential approximation for *y*(*r*) is given by

(69) $$y\left(r\right)=\frac{ze}{kT}\;\psi^{\left(0\right)}\left(r\right)=\frac{ze\zeta }{kT}\frac{a}{r}e^{-\kappa \left(r-a\right)}$$

For particles with a thin electrical double layer (*ĸa »* 1), the electric potential *Ψ*^(0)^(*r*) becomes almost zero beyond the electrical double layer at *r* = *a*+ 1/*ĸ*. In Equation (67) for $\hat{\mu }\left(s\right)$ that involves *Ψ*^(0)^(*r*), putting *r* = *a* + (*r* − *a*) makes (*r* − *a*)/*a* of the order of 1/(*ĸa*), which can be regarded as a small quantity. By expanding $\hat{\mu }\left(s\right)$ around *r* = *a* and retaining terms up to the order of ((*r* − *a*)/*a*)^2^, we obtain the following large-*ĸa* approximate form for $\hat{\mu }\left(s\right)$ for *ĸa* » 1:

(70) $$\;\hat{\mu }\left(s\right)=\frac{1+a\sqrt{\lambda^{2}+\frac{s}{\nu }}}{3s\left\{1+a\sqrt{\lambda^{2}+\frac{s}{\nu }}+\frac{\left(\lambda^{2}+\frac{s}{\nu }\right)a^{2}}{9}+\frac{2\rho_{p}}{9\rho_{o}}\;\frac{a^{2}}{\nu }s\right\}}\int_{a}^{\infty }\left(r-a^{2}\right)G\left(r\right)dr$$

where the relation $\hat{G}\left(r,s\right)=G\left(r\right)/s$ (Equation (68)) has been used. 

From Equation (69), we obtain the following large-*ĸa* approximate expression for the steady-state gel diffusiophoretic mobility *μ*(*∞*) using the relation $\mu \left(\infty \right)=\underset{s\to 0}{\lim s}\hat{\mu }\left(s\right)$:

(71) $$\mu \left(\infty \right)=\frac{1+\lambda a\;}{3\left(1+\lambda a+\frac{\lambda^{2}a^{2}}{9}\right)}\overset{\infty }{\underset{a}{\int }}{\left(r-a\right)}^{2}G\left(r\right)dr$$

Here the large-*ĸa* approximate expression of *μ*(*∞*) correct to the order of *ζ*^2^ has been derived in Ref. [52] as

(72) $$\mu \left(\infty \right)=\frac{\varepsilon_{r}\varepsilon_{0}\left(1+\lambda a\right)}{\eta \left(1+\lambda a+\frac{\lambda^{2}a^{2}}{9}\right)}\left\{\beta \zeta +\frac{kT}{8ze}{\left(\frac{ze\zeta }{kT}\right)}^{2}\right\}$$

By combining Equations (70) and (72), we obtain

(73) $$\hat{\mu }\left(s\right)=\frac{\left(1+\lambda a+\frac{\lambda^{2}a^{2}}{9}\right)\left(1+a\sqrt{\lambda^{2}+\frac{s}{\nu }}\right)\;}{s\left(1+\lambda a\right)\left\{1+a\sqrt{\lambda^{2}+\frac{s}{\nu }}+\frac{\left(\lambda^{2}+\frac{s}{\nu }\right)a^{2}}{9}+\frac{2\rho_{p}}{9\rho_{o}}\;\frac{a^{2}}{\nu }s\right\}}\mu \left(\infty \right)$$

Applying the inverse Laplace transform $L^{-1}$ to Equation (73) yields

(74) $$\mu \left(t\right)=R\left(t\right)\mu \left(\infty \right)$$

where:

(75) $$R\left(t\right)=L^{-1}\left[\frac{\left(1+\lambda a+\frac{\lambda^{2}a^{2}}{9}\right)\left(1+a\sqrt{\lambda^{2}+\frac{s}{\nu }}\right)\;}{s\left(1+\lambda a\right)\left\{1+a\sqrt{\lambda^{2}+\frac{s}{\nu }}+\frac{\left(\lambda^{2}+\frac{s}{\nu }\right)a^{2}}{9}+\frac{2\rho_{p}}{9\rho_{o}}\;\frac{a^{2}}{\nu }s\right\}}\right]\;\;$$

which can be estimated numerically. Equation (74), combined with Equation (75), is the required expression for the transient gel diffusiophoretic mobility *μ*(*t*).

## 3. Results and Discussion

In the present paper, we have considered the time-dependent transient gel diffusiophoresis of a charged spherical colloidal particle. Compared to electrophoresis, diffusiophoresis has the advantage of inducing particle motion without requiring an external electric field, making it applicable in situations where electric fields may be undesirable. Furthermore, compared to free-solution diffusiophoresis, gel diffusiophoresis offers better control over particle transport due to the constrained environment provided by the gel matrix. Additionally, while steady-state gel diffusiophoresis describes the long-term motion of particles under a constant concentration gradient, transient gel diffusiophoresis captures the dynamic response of particles to a suddenly applied electrolyte concentration gradient, which is essential for understanding time-dependent behaviors in complex environments.

To the best of our knowledge, no experimental studies on transient gel diffusiophoresis have been reported. Therefore, we propose the following experimental approach to validate the theoretical predictions: Experimentally, suddenly applying an electrolyte concentration gradient to a suspension of colloidal particles, as described by Equation (1), for transient gel diffusiophoresis is significantly more difficult than suddenly applying an electric field for transient electrophoresis. A possible method is to first establish an electrolyte concentration gradient in a polymer gel medium containing an electrolyte solution and then introduce colloidal particles into the system at *t*=0. This approach may allow for controlled observation of transient gel diffusiophoresis and provide experimental validation of the theoretical model.

We derived a closed-form approximate expression for the inverse Laplace transform of the transient gel diffusiophoretic mobility, *μ*(*t*), of a weakly charged spherical colloidal particle with a thin electrical double layer (*ĸa* » 1), given by Equation (74) as combined with Equation (54). It can be shown that, under conditions of low zeta potential and thin electrical double layer (*ĸa* » 1), the approximate expression for the transient gel diffusiophoretic mobility takes the same form as that for the transient gel electrophoretic mobility [75]. However, it should be noted that despite the similarity between transient gel diffusiophoresis and gel electrophoresis, the steady-state mobility of *μ*(*∞*) is different between gel diffusiophoresis and gel electrophoresis, reflecting different driving forces. In the case of the gel electrophoresis of a weakly charged spherical colloidal particle with a thin electrical double layer, *μ*(*∞*) is given by [75],

(76) $$\mu \left(\infty \right)=\frac{\varepsilon_{r}\varepsilon_{0}\left(1+\lambda a\right)}{\eta \left(1+\lambda a+\frac{\lambda^{2}a^{2}}{9}\right)}\zeta$$

The function *R*(*t*) given by Equation (75) can be interpreted as a relaxation function, which describes how the transient gel diffusiophoretic mobility *μ*(*t*) changes from its initial value of zero to its final steady-state value *μ*(*∞*). As mentioned above, *R*(*t*) takes the same form for the transient gel electrophoresis and diffusionphoresis. It is important to note that *R*(*t*) does not depend on the ionic drag coefficients *λ_±_*, making it independent of the specific electrolyte type (e.g., KCl or NaCl). On the other hand, *μ*(*∞*) depends on the specific electrolyte type, as shown in Equation (72) through the parameter *β* defined by Equation (40).

**Figure 2:**
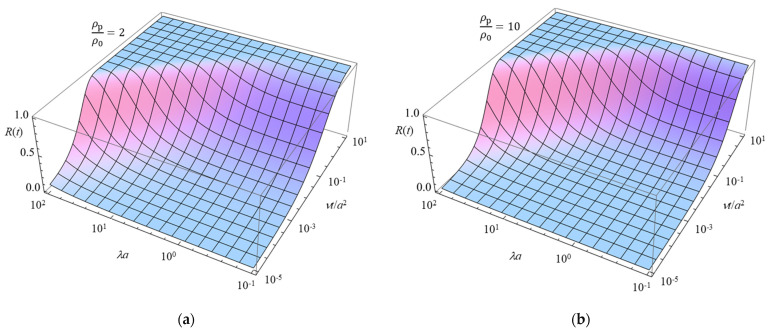
Relaxation function *R*(*t*) for the transient gel diffusiophoresis of a spherical colloidal particle with radius *a* and a thin electrical double layer (*ĸa* » 1), defined as *R*(*t*) = *μ*(*t*)/*μ*(*∞*). The 3D plot of *R*(*t*) is shown as a function of *λa, λ* being the Brinkman parameter*,* and the scaled time *νt/a*^2^, calculated using Equation (75) for *ρ*_p_/*ρ*_0_ = 2 (**a**) and 10 (**b**).

Figure 2 presents a 3D plot of *R*(*t*) as a function of the scaled particle radius *λa* and the scaled time *νt*/*a*^2^, calculated using Equation (75) for two values of the particle-to-solution mass density ratio *ρ*_p_/*ρ*_0_, that is, *ρ*_p_/*ρ*_0_ = 2 (**a**) and 10 (**b**).

Figure 2 illustrates how the transient response of particle mobility is influenced by the mass density ratio *ρ*_p_ *ρ*_0_. Heavier particles (*ρ*_p_/*ρ*_0_ = 10) exhibit a slower relaxation toward their steady-state mobility *μ*(∞), requiring a longer time to approach equilibrium. This trend is consistent with the theoretical model, as the particle inertia becomes more significant at higher mass density ratios, leading to a delayed response to the imposed electrolyte concentration gradient.

Moreover, Figure 2 demonstrates that the relaxation time required for *μ*(*t*) to reach its steady-state value *μ*(∞) decreases as *λa* increases. This behavior can be explained by Equation (72) for *μ*(∞), which shows that *μ*(∞) itself decreases with increasing *λa*. An increase in *λa* corresponds to either a larger Brinkman parameter *λ* or a larger particle radius *a*, both of which enhance the hydrodynamic resistance exerted by the polymer segments in the gel. As a result, the particle experiences greater resistance from the polymer matrix, making it more difficult to migrate through the gel, thereby leading to a reduction in its steady-state diffusiophoretic mobility *μ*(∞). As a result, the difference between the initial and steady-state mobility becomes smaller for larger *λa*, leading to a shorter relaxation time. This finding highlights the role of hydrodynamic interactions in gel diffusiophoresis, where the Brinkman parameter λ characterizes the extent of the fluid velocity field penetration into the gel network. A higher *λa* corresponds to stronger hydrodynamic screening, which reduces the overall mobility and accelerates the transient response. Thus, Figure 2 provides insight into how both the mass density ratio and the Brinkman parameter influence the time-dependent behavior of transient gel diffusiophoresis, agreeing with the theoretical predictions.

The present study demonstrates that while the steady-state mobility *μ*(∞) is primarily a function of the scaled particle radius *λa* and the zeta potential *ζ*, the transient mobility *μ*(*t*) exhibits additional dependencies on the scaled time *νt*/*a*^2^ and the mass density ratio *ρ*_p_/*ρ*_0_. This highlights a fundamental distinction between transient diffusiophoresis in gels and traditional gel electrophoresis, where transient effects are often neglected. The dependence of *μ*(*t*) on *ρ*_p_/*ρ*_0_ suggests that transient mobility carries information about particle inertia, which is absent in the steady-state analysis. Thus, analyzing *μ*(*t*) provides a more comprehensive understanding of particle motion in gels, offering deeper insights into the underlying transport mechanisms compared to steady-state mobility alone.

For the case of large *t*, which corresponds to small *s* in Equation (73), the inverse Laplace transform of Equation (75) can be derived analytically as follows. For small *s*, Equation (73) reduces to

(77) $$\hat{\mu }\left(s\right)=\frac{1+\lambda a+\frac{\lambda^{2}a^{2}}{9}}{s\left(1+\lambda a+\frac{\lambda^{2}a^{2}}{9}+\frac{2\rho_{p}}{9\rho_{o}}\;\frac{a^{2}}{\nu }s\right)}\mu \left(\infty \right)$$

from which we obtain

(78) $$\mu \left(t\right)=\left(1-e^{-t/T}\right)\mu \left(\infty \right)\;$$

or

(79) $$R\left(t\right)=1-e^{-t/T}\;$$

with

(80) $$T=\frac{2}{9\left(1+\lambda a+\frac{\lambda^{2}a^{2}}{9}\right)}\left(\frac{\rho_{p}}{\rho_{0}}\right)\left(\frac{a^{2}}{\nu }\right)\;$$

Here *T* can be regarded as the relaxation time. which describes how *T* depends on *λa*, and *ρ*_p_/*ρ*_0_, and *νt*/*a*^2^. The relaxation time *T* for the transient gel diffusiophoresis is shorter than the relaxation time *T* for the transient free-solution gel diffusiophoresis (with *λ* = 0) by a factor 1 + *λa* + *λ*^2^*a*^2^/9. This shows that *T* decreases as *λa* increases.

## 4. Conclusions

We have developed a theory of the transient gel diffusiophoresis of a charged spherical colloidal particle in an uncharged polymer gel medium, subjected to sudden electrolyte concentration gradients. The relaxation function *R*(*t*) (Equation (75)) characterizes the time-dependent gel diffusiophoretic mobility of a weakly charged spherical colloidal particle with a thin electrical double layer (*ĸa* » 1). Our results show that *R*(*t*) is determined by the particle-to-solution mass density ratio *ρ*_p_/*ρ*_0_, the Brinkmann screening parameter *λ*, the particle radius *a*, and the kinematic viscosity *ν* of the solution, while it does not depend on the specific electrolyte type (e.g., KCl or NaCl), which only affects the steady-state gel diffusiophoretic mobility (Equation (72)).

## Data Availability

Data are contained within the article.
